# Supplementary material for: Therapeutic Efficacy of GC1118, a Novel Anti-EGFR Antibody, against Glioblastoma with High EGFR Amplification in Patient-Derived Xenografts
Source: Cancers (Basel). 2020 Oct 31;12(11):3210. doi: 10.3390/cancers12113210 (PMC7693807; doi:10.3390/cancers12113210)
Supplement: Supplementary file 1 [file cancers-12-03210-s001.pdf]

Supplementary Material: Therapeutic Efficacy of Gc1118, a Novel Anti-Egfr Antibody, against Glioblastoma with High Egfr Amplification in Patient-Derived Xenografts

Kyoungmin Lee, Harim Koo, Yejin Kim, Donggeon Kim, Eunju Son, Heekyoung Yang, Yangmi Lim, Minkyu Hur, Hye Won Lee, Seung Won Choi and Do-hyun Nam

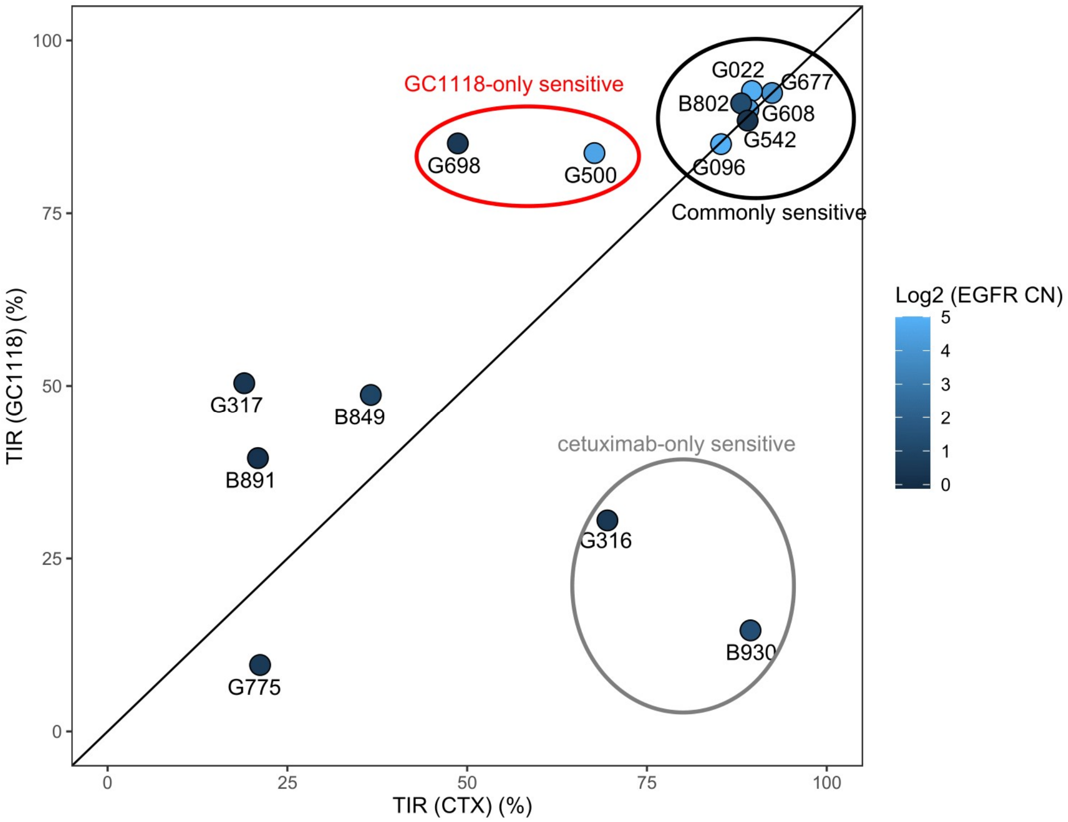

Figure S1. Comparison of anti-tumor effect of GC1118 and cetuximab. GBM PDXs exhibited different therapeutic vulnerability against GC1118 and cetuximab, despite their common mechanism of action (rho = 0.57, *p*-value = 0.03, Pearson correlation test). G698 and G500 were more sensitive to GC1118, while G316 and G930 exhibited specific sensitivity to cetuximab.

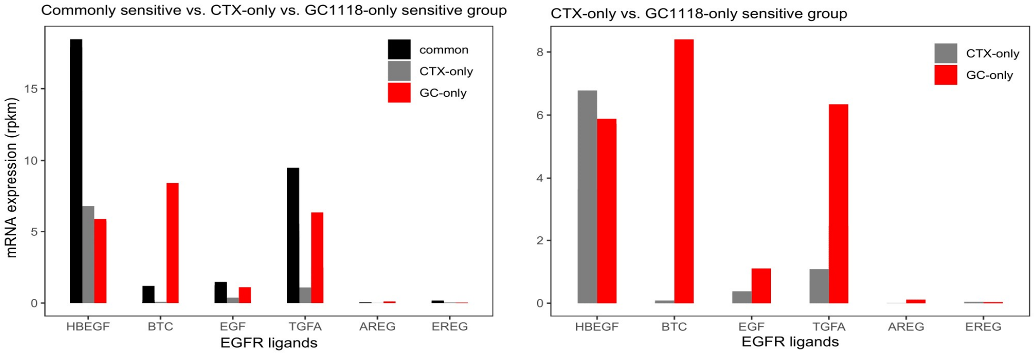

Figure S2. mRNA expression of EGFR ligands in GBM tumors. Using patient-derived transcriptomic data (n = 8), we compared the mRNA expression of EGFR ligands. EGFR ligands are classified into

two subsets, high-affinity (heparin-binding EGF-like growth factor (HB-EGF), β-cellulin (BTC), EGF, and TFGα) vs. low-affinity ligands (amphiregulin (AREG) and epiregulin (EREG)). GBM tumors exhibited predominant expression of high-affinity ligands. The expression of BTC and TGFα was upregulated in the GC1118-only sensitive group, although not statistically significant.

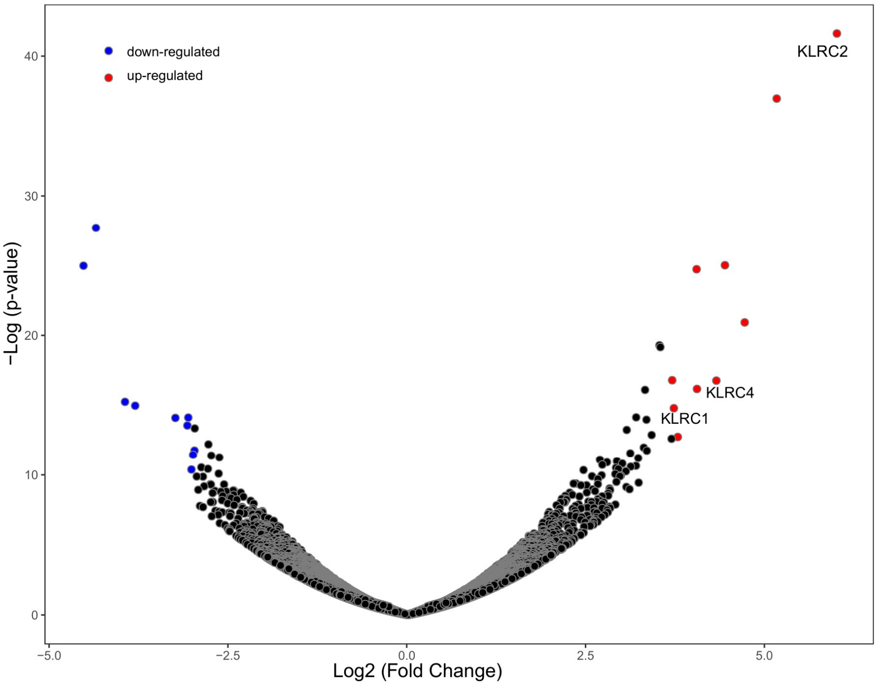

Figure S3. A volcano plot depicting differential gene expression analysis between the GC1118-only (n = 2) and cetuximab-only sensitive group (n = 2).

© 2020 by the authors. Licensee MDPI, Basel, Switzerland. This article is an open access article distributed under the terms and conditions of the Creative Commons Attribution (CC BY) license (http://creativecommons.org/licenses/by/4.0/).
